# Dynamic Digital Radiography in Ehlers–Danlos Syndrome: Visualizing Diaphragm Motility Impairment and Its Influence on Clinical Management

**DOI:** 10.3390/diagnostics15111343

**Published:** 2025-05-27

**Authors:** Elisa Calabrò, Maurizio Cè, Francesca Lucrezia Rabaiotti, Laura Macrì, Michaela Cellina

**Affiliations:** 1 Pulmonology Department, ASST Fatebenefratelli Sacco, Piazza Principessa Clotilde 3, 20121 Milan, Italy; elisa.calabro@asst-fbf-sacco.it; 2 Postgraduation School in Radiodiagnostics, Università degli Studi di Milano, Via Festa del Perdono 7, 20122 Milan, Italy; maurizioce.md1@gmail.com (M.C.); francesca.rabaiotti@unimi.it (F.L.R.); laura.macri@unimi.it (L.M.); 3 Radiology Department, ASST Fatebenefratelli Sacco, Piazza Principessa Clotilde 3, 20121 Milan, Italy

**Keywords:** dynamic digital radiography, diaphragm dysfunction, respiratory imaging

## Abstract

A 40-year-old woman with a known diagnosis of Ehlers–Danlos syndrome (EDS) began experiencing progressive shortness of breath and reduced exercise tolerance following her second pregnancy. The patient underwent an unenhanced computed tomography (CT) scan that showed a marked elevation of the left diaphragm. Suspecting diaphragm dysfunction, the patient underwent Dynamic Digital Radiography (DDR) that confirmed a reduction in left diaphragm motility, indicative of impaired diaphragm function. Based on the DDR findings, which demonstrated reduced but preserved diaphragmatic motion without paradoxical movement or complete immobility, the thoracic surgeon decided that surgical intervention, such as plication, was not necessary. Instead, rehabilitation exercises, including breathing techniques and diaphragm strengthening, were recommended. EDS includes connective tissue disorders that vary in severity but are typically characterized by hypermobility of the joints, skin hyper-elasticity, and a predisposition to vascular fragility. One of the complications of EDS is weakened connective tissues, which can affect the diaphragm, impairing the contractility of the muscle and leading to impaired mobility and respiratory symptoms such as shortness of breath. Diaphragm dysfunction can manifest as reduced movement, potentially related to the underlying connective tissue weakness. This case highlights the clinical value of DDR as a non-invasive, low-dose, and dynamic imaging modality in the diagnosis of diaphragmatic dysfunction in EDS patients, enabling individualized treatment planning and potentially avoiding unnecessary surgical interventions.

**Figure 1:**
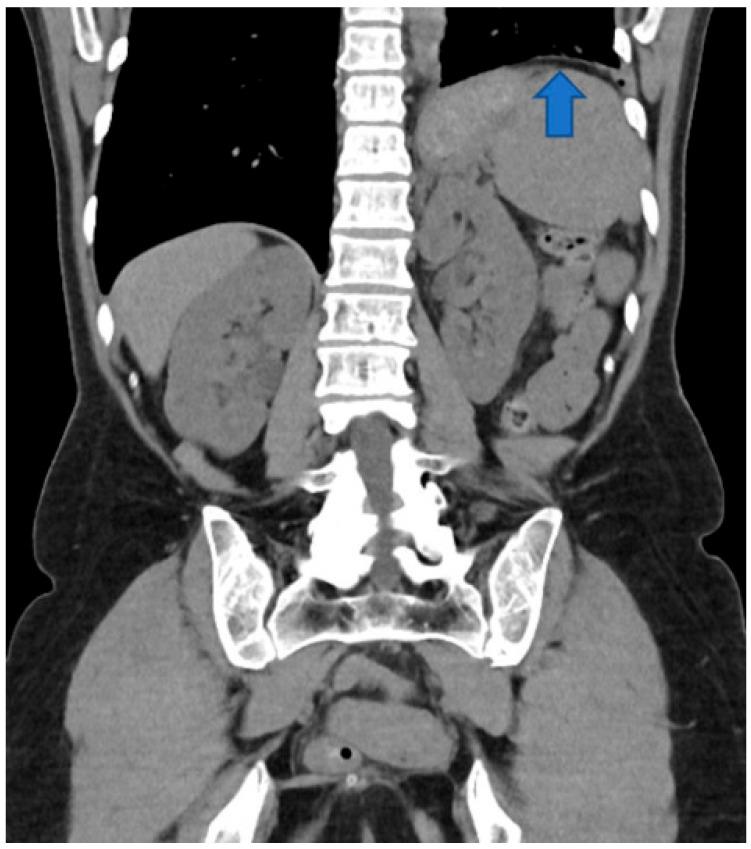
Coronal reconstruction of the unenhanced CT scan. The left diaphragm is abnormally elevated (blue arrow).

**Figure 2:**
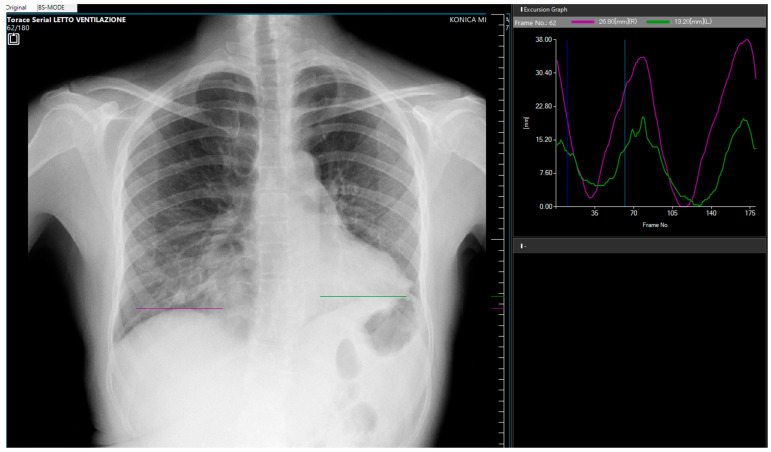
Posteroanterior Dynamic Digital Radiography (DDR) during forced breathing (AeroDR TX, Konica Minolta Inc., Tokyo, Japan). Acquisition parameters were: tube voltage, 100 kV; tube current, 50 mA; 1.6 ms; source-to-image distance, 2 m; additional filter, 0.5 mm Al +0.1 mm Cu; exposure, 12 s; 15 frames/s. The pixel size was 388 × 388 μm, the matrix size, 1024 × 768, and the overall image area 40 × 30 cm. Diaphragmatic motion was assessed using the dedicated Konica Minolta workstation software (DX-1), which allows for a dynamic review of sequential images in cine mode. The purple curve represents the right diaphragm’s motion, and the green curve represents the left diaphragm’s motion [1]. Note the reduced excursion of the green curve, indicating impaired left diaphragm motility (Supplementary Materials S1, S2). The vertical displacement of the left hemidiaphragm was visually assessed relative to the contralateral side during deep breathing. While quantitative motion analysis software is available, in this case, the assessment was based on qualitative grading of diaphragm excursion reduction compared to the contralateral (right) hemidiaphragm within the same acquisition. Diaphragm dysfunction diagnosis is quite challenging, mainly based on medical history (symptoms such as shortness of breath, especially when lying down, unexplained fatigue, or reduced exercise tolerance) and physical exam (decreased breath sounds at the lung bases) [2]. A standard X-ray can reveal an elevated hemidiaphragm, which may suggest paralysis or dysfunction. Ultrasound may allow direct visualization of the diaphragm and its motion but is strictly related to the operator’s experience. CT or MRI provide detailed anatomy but no dynamic functional assessment [3,4]. Unlike traditional static imaging, DDR provides dynamic visualization of diaphragm movement during respiration, allowing for a more accurate assessment of diaphragm motility and function [1,4]. This technique captures a series of high temporal resolution images during patient breaths, generating a motion study of the diaphragm [1]. This allows for visualization of the diaphragm’s excursion, speed, and symmetry during inspiration and expiration [2]. The use of pulsed X-ray allows a lower radiation exposure when compared to fluoroscopy [1]. The post-processing also provides information on the motility in different areas of the lungs [5]. The right lung shows normal motility, with a well-represented motion in the lower field. The motility of the left lung is instead reduce, with fewer and smaller green arrows (as shown in Supplementary Materials S2).

## Data Availability

No new data were created or analyzed in this study. Data sharing is not applicable to this article.

## Supplementary Materials

The following supporting information can be downloaded at: https://www.mdpi.com/article/10.3390/diagnostics15111343/s1, Video Supplementary Material S1: Digital Dynamic Radiography acquisition during the breath cycle showing the impaired motility of the left diaphragm; Video Supplementary Material S2: Digital Dynamic Radiography acquisition showing the reduced motility of the left lung.
